# A pre-zygotic barrier to hybridization in two con-generic species of scleractinian corals

**DOI:** 10.12688/f1000research.2-193.v2

**Published:** 2013-11-01

**Authors:** Andrew H. Baird, Vivian R. Cumbo, Joana Figueiredo, Saki Harii

**Affiliations:** 1 Australian Research Council, Centre of Excellence for Coral Reefs Studies, James Cook University, Townsville, Australia; 2 Sesoko Station, Tropical Biosphere Research Center, University of the Ryukyus, Okinawa, Japan

## Abstract

Hybridization is often cited as a potential source of evolutionary novelty in the order *Scleractinia*. While hybrid embryos can be produced *in vitro*, it has been difficult to identify adult hybrids in the wild. Here, we tested the potential for hybridization between two closely related species in the family Fungiidae. We mixed approximately 5000 eggs of *Ctenactis echinata *with sperm from *C. crassa*. No hybrid embryos were produced. This observation adds to a growing body of evidence for pre-zygotic barriers to hybridization in corals and challenges the claim that hybridization is a major source of evolutionary novelty in the order.

## Observation

Hybridization is a controversial topic in coral reef ecology ^1, 2^. While small numbers of hybrid embryos can be produced in a few species *in vitro* ^3^, the evidence for hybrids in the field is often equivocal because the genetic techniques used for corals cannot distinguish between hybridization and incomplete lineage sorting ^4^. In fact, only one of the over 1300 species in the order is generally accepted to be unequivocally of hybrid origin: *Acropora prolifera* ^1, 5^. Nonetheless, hybridization is often invoked as a source of evolutionary novelty in the order *Scleractinia* ^6, 7^.

**Figure 1.**
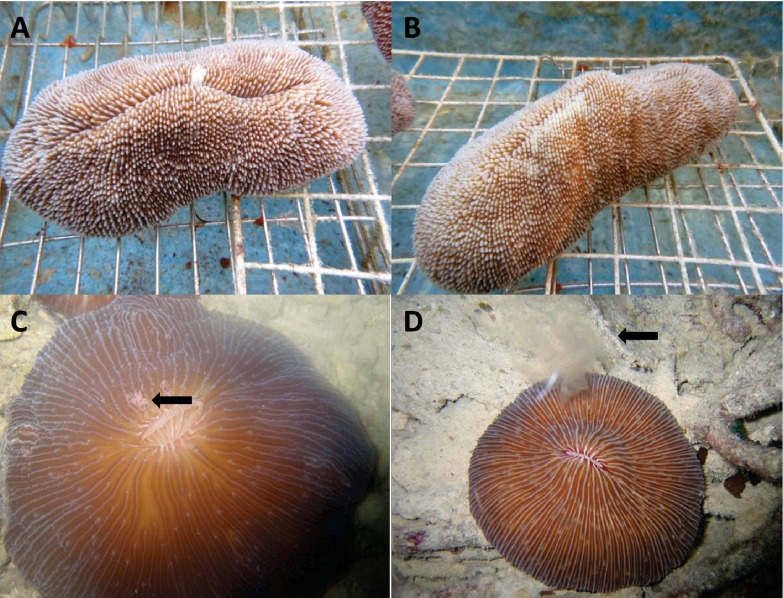
Study species and broadcast spawning in fungiid corals. Live *Ctenactis echinata* ( **A**) and *C. crassa* ( **B**) in aquaria prior to being isolated for spawning. Each colony is approximately 20 cm in length. Coral species in the family Fungiidae, such as these colonies of *Fungia fungites*, are gonochoric broadcast spawners: each individual releases either eggs ( **C**) or sperm ( **D**) into the water column where fertilization takes place (arrows indicate gametes).

Here, we report an incidental observation on the potential for hybridization between two closely related scleractinian corals species in the family Fungiidae, *Ctenactis echinata* and *C. crassa*. These species are sympatric, often dominating large multi-specific assemblages of fungiid corals throughout the central Indo-Pacific ^8^. These species can generally be distinguished on the basis of the density of septa and the shape of septal dentitions, however, in Okinawa, these features are very similar and the most useful diagnostic character is a strong arch in the corallum of *C. crassa* ( Figure 1A and B) ^9^. Both species are gonochoric, that is each colony is either male or female, and reproduce by broadcast spawning, releasing gametes into the water column for fertilization ^8^ ( Figure 1C and D). At our study site on Sesoko Island (26°38'13.00"N; 127°51'56.24"E), Okinawa, Japan, spawning occurs following the full moons from July to August ^8^. Furthermore, both species release gametes at the same time ^8^ and consequently there is the potential for hybridization. In the days before the predicted date of spawning in July 2013, we collected four colonies of *C. echinata* and six colonies of *C. crassa*, to produce larvae for other experiments.

While the species are relatively easy to identify, determining the sex of each individual prior to spawning is impossible without destructive sampling to expose the gametes. Consequently, we placed each individual in a separate 20 L bucket containing sea water in the open air at approximately 20:00 h in order to sex each individual once gametes had been released. On the night of 27 July between 22:30 and 23:30 h three *C. echinata* and five *C. crassa* spawned revealing that the three spawning *C. echinata* were female, while four *C. crassa* were females and one was a male. The size of the eggs of each species at the time of release was distinct with a range in maximum diameter of 244–266 μm in *C. echinata* and 133–155 μm in *C. crassa*. In contrast to earlier work on *C. echinata* ^10^, we saw no symbiotic algae in the eggs of either species. We collected approximately 5000 eggs from the three *C. echinata* females and mixed them with sperm from the *C. crassa* male. The viability of the *C. crassa* sperm was tested by mixing it with *C. crassa* eggs, however, we could not quantify the viability of the *C. echinata* eggs because no *C. echinata* sperm was available on the evening of the experiment. Nonetheless, eggs from these colonies of *C. echinata* did produce viable larvae for use in later experiments. Approximately 100 eggs were observed under a stereo-dissecting microscope for cleavage, indicating fertilization, every 2 to 6 h over the next 24 h. At no point did we observe cleavage in the cross between species indicating that no hybrid embryos were produced and none of the approximately 5000 eggs remained intact after 24 h. In contrast, over 90% of *C. crassa* eggs in the positive control were fertilized within 2 h. We conclude that despite synchrony in the time of gamete release between these two closely related sympatric species there appears to be strong pre-zygotic mechanism to avoid hybridization. While our observations are preliminary and in only one direction (i.e. we did not cross *C. echinata* males with *C. crassa* females) we predict that hybridization between these species is unlikely. This observation adds to a growing body of evidence indicating strong pre-zygotic barriers to hybridization in many scleractinian corals ^11– 13^.
