# Supplementary material for: A pre-zygotic barrier to hybridization in two con-generic species of scleractinian corals
Source: F1000Res. 2013 Nov 1;2:193. Originally published 2013 Sep 20. [Version 2] doi: 10.12688/f1000research.2-193.v2 (PMC4647700; doi:10.12688/f1000research.2-193.v2)
Supplement: Peer review history

# Peer review: Referee response for version 2

**Published:** 2014-01-23

**Authors:** Bernie Degnan

**Affiliations:** 1 School of Biological Sciences, University of Queensland, Brisbane, Australia

I have read this submission. I believe that I have an appropriate level of expertise to confirm that it is of an acceptable scientific standard.

# Peer review: Referee response for version 2

**Published:** 2013-11-13

**Authors:** Yossi Loya

**Affiliations:** 1 Department of Zoology, Tel Aviv University, Tel Aviv, Israel

I note that the authors preferred to retain Figures 1C and 1D in spite of my recommendation to delete them, since they have nothing to do with the contents of the article. I do not insist that they do it, but it may confuse the readers. I wonder though where did the photos of *Fungia fungites* were taken? I assume the photos were taken from a GBR specimen, since this species is a brooder and not a broadcaster in Okinawa.

I have read this submission. I believe that I have an appropriate level of expertise to confirm that it is of an acceptable scientific standard.

# Peer review: Referee response for version 1

**Published:** 2013-10-24

**Authors:** Bernie Degnan

**Affiliations:** 1 School of Biological Sciences, University of Queensland, Brisbane, Australia

This Observation Article reports the lack of cross-fertilization between *Ctenactis echinata* and *Ctenactis crassa*, closely related fungiid corals that naturally release gametes at the same time. The authors recognise the limitations of this observation - only small numbers of eggs (100's) were observed and only eggs from *C. echinata *were available to be fertilized (i.e. they did not cross *C. echinata* males with *C. crassa* females) - but rightly point out that this study provides further evidence that hybridization is not as widespread amongst scleractinian corals as often portrayed in the literature. However, the analysis of reciprocal crosses and a larger numbers of eggs is necessary before it can be said with some confidence that these congeners can not hybridize.

I have read this submission. I believe that I have an appropriate level of expertise to confirm that it is of an acceptable scientific standard.

# Response

**Published:** 2013-10-26

**Authors:** Andrew Baird

**Affiliations:** School of Marine Biology and Aquaculture, James Cook University, Australia

Dear Bernie,

Thank you for your comments. We would just like to point out that while we only examined 100 eggs every few hours under the microscope, we used approximately 5000 eggs in the fertilization experiment, all of which had broken down after 24 h suggesting none had fertilized. We have added a sentence to the revised text to draw attention to this.

# Peer review: Referee response for version 1

**Published:** 2013-10-22

**Authors:** Yossi Loya

**Affiliations:** 1 Department of Zoology, Tel Aviv University, Tel Aviv, Israel

- The title is appropriate for the content of the article. The abstract represents a suitable summary of the work. Please correct: *crass *to *crassa*, in the 3 ^rd^^ ^line of the Abstract.
   
- Article content: The design, methods and analysis of the results been clearly explained and are appropriate for the topic being studied. Figures 1C and 1D appear to be irrelevant to the article since they show different species. I suggest deleting them. A proper reference to the statement *'spawning occurs following the full moons from July to August'* (reference 8 in the manuscript) is: Loya Y. & K. Sakai (2008). Bidirectional sex change in mushroom corals. *Proc. Roy. Soc. Biol.* B 275: 2335-2343.
   
- Data and Conclusions: The authors note that they did not cross *C. echinata *males with *C. crassa* females; however they also did not test the positive control of crossing *C. echinata* males with *C. echinata* females (all their experimental *C. echinata *specimen were females). Nevertheless, this does not diminish their prediction that hybridization between these species is unlikely. The paper contributes further information to the controversial topic of potential hybridization and breeding incompatibilities within the mating systems of broadcast spawning reef corals.

I have read this submission. I believe that I have an appropriate level of expertise to confirm that it is of an acceptable scientific standard.

# Response

**Published:** 2013-10-26

**Authors:** Andrew Baird

**Affiliations:** School of Marine Biology and Aquaculture, James Cook University, Australia

Dear Yossi,

Thank you for your comments. We have corrected the typos you identified, changed the reference as requested and added a sentence to clarify the controls that were used to test for gamete viability. Images 1 C & D are presented as an example of the spawning behavior of fungiids.
